# Control of gene expression through the nonsense-mediated RNA decay pathway

**DOI:** 10.1186/s13578-017-0153-7

**Published:** 2017-05-19

**Authors:** Andrew Nickless, Julie M. Bailis, Zhongsheng You

**Affiliations:** 1 Department of Cell Biology & Physiology, Washington University School of Medicine, Campus Box 8228, 660 S. Euclid Ave., St. Louis, MO 63110 USA; 2 Department of Oncology Research, Amgen, South San Francisco, CA 94080 USA

**Keywords:** Nonsense-mediated decay, RNA surveillance, Gene expression, Human disease

## Abstract

Nonsense-mediated RNA decay (NMD) was originally discovered as a cellular surveillance pathway that safeguards the quality of mRNA transcripts in eukaryotic cells. In its canonical function, NMD prevents translation of mutant mRNAs harboring premature termination codons (PTCs) by targeting them for degradation. However, recent studies have shown that NMD has a much broader role in gene expression by regulating the stability of many normal transcripts. In this review, we discuss the function of NMD in normal physiological processes, its dynamic regulation by developmental and environmental cues, and its association with human disease.

## Background

Nonsense-mediated RNA decay (NMD) is an essential RNA quality control and gene regulatory mechanism that is conserved among eukaryotes [1–9]. NMD safeguards the quality of the transcriptome and maintains cellular homeostasis by eliminating transcripts that harbor premature termination codons (PTCs). PTCs can arise from errors in nucleic acid metabolism, such as genetic mutations or defects in splicing or transcription. In addition, PTCs may also form during mRNA synthesis from normal gene structures, including from programmed recombination. In its canonical role, NMD prevents translation of transcripts that might produce C-terminally truncated proteins with reduced or aberrant function.

NMD also targets non-mutant transcripts, and its regulation of normal gene expression impacts a wide range of physiological processes including cell differentiation, response to stress and development of disease. Recent estimates suggest that NMD-mediated degradation affects up to 25% of transcripts, either directly or indirectly in certain cellular milieus [1, 10, 11]. Although the overall process of NMD is conserved for both mutant and non-mutant transcripts, the signals that trigger NMD or its inhibition vary according to the specific target and biological context.

In this review we focus on the function and impact of NMD on normal gene expression in mammals. We outline the NMD process and highlight the known roles for NMD in normal physiology, with a particular emphasis on its function as a gene regulatory mechanism and its dynamic regulation by environmental and developmental signals. We conclude with an overview of the impact of NMD dysregulation on human disease and discuss the potential of treating genetic and neurological disorders and cancer by manipulating NMD activity.

## Overview of the NMD pathway

First discovered in yeast and then extensively studied in *Caenorhabditis elegans*, *Drosophila*, mouse, human cells, and other model systems, NMD is a RNA surveillance pathway that acts at the interface between transcription and translation [10, 12–15]. NMD must accurately distinguish a PTC from a normal stop codon on an mRNA and then recruit and activate enzymes to destroy the transcript. There are two main models to explain how transcripts are identified as targets for NMD. The exon-junction complex (EJC) model proposes that EJC—a large multi-protein assembly deposited ~20–24 bases upstream of an exon–exon junction as a result of pre-mRNA splicing—acts as a second signal to mark an upstream stop codon as a PTC [16–29]. During translation, ribosomes scan the mRNA and will pause at a stop codon. If an EJC is present more than 50–55 bases downstream of the stop codon, the protein kinase SMG1, its substrate Upf1, a ATPase/helicase, and eukaryotic polypeptide release factors eRF1 and eRF3 are then recruited to form a complex—the SURF complex—on the mRNA. Phosphorylation of UPF1 by SMG1 leads to the recruitment of SMG5, SMG6 and SMG7 via phospho-specific interactions [30, 31]. After recruitment, SMG5 and SMG7 promote RNA decapping and deadenylation by recruiting factors such as DCP1a and POP2, leading to the exposure of the transcript ends to cellular exonucleases [32–39]. SMG6, which has endonuclease activity, provides a second mechanism for initiation of mRNA decay by cleaving transcripts internally near the PTC, generating two unprotected RNA ends that are further degraded by cellular nucleases [40–43].

The second model for NMD posits that the abnormally long 3′ untranslated region (UTR) downstream of a PTC acts as a second signal to promote PTC recognition. While the molecular mechanism of this model is less well defined, it has been proposed that accumulation of UPF1 as well as other regulatory elements in the 3′ UTR mediates the recruitment of other NMD factors and the initiation of mRNA decay [44–47]. For additional information about the mechanisms of PTC recognition, readers are directed to a number of recent reviews [13, 48–50].

A debate remains about where NMD takes place in the cell. NMD inherently relies on the translation process, which normally occurs in the cytoplasm. However, some investigators have proposed that translation can also take place in the cell nucleus [51–53]. Several studies suggest that NMD is associated with the nucleus or nuclear fraction. For example, levels of PTC-containing triosephosphate isomerase (TPI) and mouse major urinary protein (MUP) transcripts were specifically reduced in the nuclear fraction [54–57]. NMD-mediated degradation of the TCRβ transcript can also take place in purified nuclei [58]. Several other nonsense reporter mRNAs also appear to be targeted by NMD in the nucleus [51, 59–64]. The idea of PTC recognition in the nucleus is also consistent with the existence of nonsense-associated altered splicing (NAS), a NMD-related nuclear pathway that also requires PTC recognition [65]. However, the claims of nuclear NMD and translation remain controversial as other lines of evidence suggest that NMD is primarily a cytoplasmic process [25]. Indeed, alternative interpretations of the nuclear NMD data are possible, including the possibility that NMD occurs during nuclear export, of which there is some evidence [66].

## Role of NMD and its regulation in normal physiological processes

Bioinformatics analysis of EST databases and RNA sequencing data in cells where NMD is disrupted have clearly demonstrated that NMD has a widespread effect on gene expression [10, 67, 68]. This realization has led to the identification of numerous putative NMD target mRNAs, based on characteristics such as PTCs or long 3′ UTRs and an observed increase in the stability and/or levels of transcripts after NMD suppression [1, 4, 5, 11, 69–75].

**Fig. 1:**
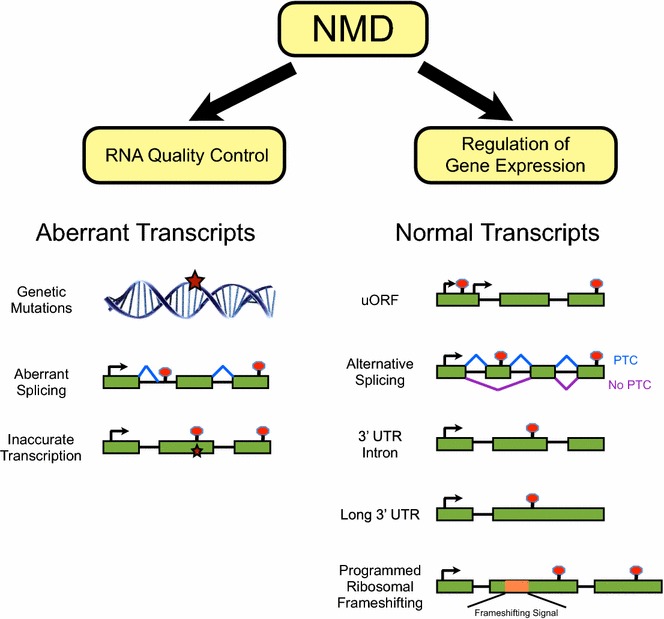
NMD functions both in RNA surveillance and in gene regulation. Several features of mRNA transcripts can mark the transcripts as substrates for degradation by NMD. *Green boxes*, exons; *lines*, introns; potential splicing events are shown by *blue* or *purple lines*; genetic mutations and transcriptional aberrations are denoted with *stars*. The translation start site (ATG) is marked by *arrows* and stop codons are marked by *red circles*

Multiple mechanisms exist to generate PTCs in transcripts of normal genes (Fig. 1). Alternative splicing generates diversity in mRNA isoforms but can also lead to formation of PTCs that target transcripts for NMD. For instance, the RNA binding protein polypyrimidine tract binding protein 1 (PTBP1) can repress splicing of exon 11 of its own mRNA, leading to NMD of the transcript [76]. As such, PTBP1 negatively regulates its own expression. Human arginine–serine rich (SR) splicing factors have also been shown to be regulated by alternative splicing coupled to NMD [77, 78]. This so-called unproductive splicing and translation (RUST) represents an autoregulatory mechanism that controls the levels of splicing factors and other RNA binding proteins [77–81]. The use of alternative transcription initiation sites can also generate mRNA isoforms with a stop codon upstream of a splice junction, resulting in NMD [82]. Programmed ribosomal frameshifting (PRF)—which can potentially occur in up to 8–12% of genes—is another mechanism that can create a PTC, leading to NMD [83–86]. Stop codons that trigger NMD can also form if the primary coding region of an mRNA is preceded by an upstream open reading frame (uORF) [87, 88]. Transcriptome analysis indicates that long 3′ UTRs are among the most common features of NMD targets, although 3′ UTR length per se is not considered a reliable predictor of NMD of a given transcript [29, 44–46, 74, 88–91].

Transcripts encoding selenoproteins comprise another interesting class of NMD targets. A UGA codon normally signals a stop to translation but can be redefined to code for the amino acid selenocysteine in a high selenium environment [92]. If selenocysteine is incorporated in the last exon of a transcript it generally evades NMD, while if selenium is not abundant, these transcripts will be degraded via NMD if their stop codon resides upstream of an exon–exon junction [93, 94]. This regulatory mechanism enables cells to respond to alterations in levels of the essential trace element selenium. Therefore, while some NMD targets—such as those encoding selenoproteins or splicing factors—have been well characterized, the validation of other putative NMD targets is ongoing, as is the understanding of the consequences of NMD-induced regulation of gene expression.

There are many examples where normal physiological processes employ NMD to regulate gene expression (Table 1). As an example of the essential nature of NMD for normal cellular processes, knockouts of Smg1, Smg6, Upf1, or Upf2 have been shown to cause embryonic lethality in mice [1–4, 6]. NMD has also been shown to play a central role in the development and differentiation of specific cell types through regulation of gene expression.

**Table 1:** Physiological functions of NMD and its regulation in response to developmental and stress signals

Cellular/molecular processes	NMD function and regulation	Target genes	References
Suppression of aberrant transcripts and transcriptional “noise”	Targets mis-spliced or aberrantly transcribed RNAs, and those derived from mutant genes, transposons and retroviral DNA, for degradation	Many	[10, 49, 176]
Lymphocyte development	Suppresses expression of unproductive rearrangements of immunoglobulin and Tcell receptor genes	Immunoglobulin, T-cell receptors	[5, 96, 97]
Embryonic stem cell differentiation	Promotes differentiation by regulating c-myc and the TGFβ/BMP pathways	C-Myc, Smad7	[6, 99, 100]
Neurogenesis	Facilitates expression of neuron-specific NMD targets in differentiating neuronal stem cells through miR-128-mediated downregulation of NMD	Many (e.g. Enpp2, Apoe, Abca1, Atp1a2, Kcnj10, Kcnd2, Ttyh1, Ppp2rb2, Stat3, Smad5, Chrdl1, Myt1, Pla2g7, Cercam, Dmd, Slc6a13)	[90]
Myogenesis	Facilitates expression of the NMD target myogenin because increased SMD activity leads to reduced NMD function	Myogenin	[112]
Cellular viability	Suppresses expression of GADD45α, leading to inhibition of apoptosis	GADD45α	[140]
lncRNA regulation	May influence micropeptide expression from a subset of lncRNA transcripts	~17% of lncRNAs	[129, 130]
Granulocyte differentiation	Suppresses expression of genes that control granulocyte differentiation and morphology	Dozens (e.g. Lmnb1)	[98]
Axon guidance	Guides axon migration by limiting the expression of Robo3.2 in commissural neurons	Robo3.2	[109]
Synaptic regulation	Impacts the expression of synaptic genes regulated by the RNA-binding protein NOVA, which is itself regulated by synaptic activity	Many (e.g. Dlg3, Dzip1, Ahi1, Slc4a3, Slc4a10, Rasgrf1, Act16b, Scn9a, Stx2, Cdk5rap2, Stxbp2, Plekha5, Lrrcc1)	[110]
Response to viral infection	Targets viral RNAs to reduce viral load, but also downregulated by specific RNA elements or protein products (e.g. Tax and Rex) to protect viral RNAs	Gag in RSV, HTLV-1 RNAs	[114–119]
Stress response (e.g. amino acid deprivation, hypoxia, ER stress)	Upregulates stress response genes as a consequence of downregulation of NMD activity by eIF2α phosphorylation	Many (e.g. ATF4, ATF3, CHOP, IRE1α)	[70, 73, 131–136]
Response to chemotherapeutics	Upregulates pro-apoptotic NMD target genes, as a consequence of UPF1 cleavage by caspases during early stages of apoptosis that downregulates NMD	GADD45α, GADD45β, BAK1, GAS5, DAP3, DUSP2	[138, 139]

During lymphocyte development, cells undergo a series of programmed genomic rearrangements to assemble immunoglobin and T cell receptor (TCR) genes. Two-thirds of these rearrangement events yield unproductive gene products harboring PTCs, whose clearance requires NMD [5, 95, 96]. Consistent with this observation, conditional ablation of NMD in T-cells significantly increased the abundance of these nonsense TCR transcripts, resulting in apoptotic cell death [5]. Interestingly, thymocyte development could be restored by introducing a complete TCRβ sequence that prevents the accumulation of nonsense counterparts, indicating that removal of the mutant transcripts by NMD is key to the survival of these cells [97]. However, conditional knockout of Upf2 had minimal effects on mature T cells, perhaps because T cells naturally downregulate NMD as part of the differentiation process [5]. In the myeloid lineage, the LMNB1 mRNA is specifically downregulated by NMD during granulopoiesis due to programmed intron retention. Importantly, this regulation of LMNB1 mRNA is required for normal differentiation of granulocytes [98].

Similar to the hematopoietic system, embryonic stem cells (ESCs) rely on NMD for their proliferation, while their differentiation is associated with downregulation of NMD activity [99, 100]. NMD influences stem cell differentiation by regulating the signaling of two key growth factors, TGFβ and BMP [100]. Ablation of NMD by SMG6 knockout in mouse ESCs prevented cellular differentiation, and re-expression of wild type but not mutant SMG6 restored proper differentiation [6]. Knockdown of other NMD factors caused a similar phenotype [6]. Prolonged, elevated expression of NMD-regulated pluripotency genes, such as c-Myc, underlies the inability of NMD-deficient ESCs to differentiate [6].

A number of studies have also revealed connections between NMD and proper development of the nervous system [90, 101–109]. In mammals, UPF3B expression is altered during brain development, and UPF3B mutants with impaired NMD function inhibit proper neurite outgrowth [105, 107]. Neuronal development is also compromised when UPF3B is downregulated with shRNA or when NMD is inhibited with the compound Amlexanox [105, 107]. NMD also functions to limit the expression of Robo3.2 in commissural neurons, which is required for proper axonal migration during development [109]. NMD components such as SMG1, UPF1 and UPF2 can localize to axonal growth cones in neurons, consistent with the idea that NMD modulates gene expression locally in these structures [109]. During the process of neurogenesis, a regulatory circuit is activated in differentiating neurons whereby expression of miR-128 targets mRNAs of several NMD factors for translational repression [90]. This results in NMD attenuation and upregulation of NMD target genes, many of which foster proper neuronal development [90].

Nonsense-mediated RNA decay also can impact gene expression in mature neurons, however. Ablation of the EJC factor eIF4AIII in mature neurons results in altered expression of critical factors such as ARC, leading to increased synaptic strength [108]. During seizure, when neuronal activity is aberrant, the RNA splicing protein NOVA regulates insertion of cryptic exons into the mRNA of a number of neuronal factors, leading to NMD of these transcripts [110]. Together, these findings highlight the importance of NMD in the development and function of the nervous system.

In developing muscle cells, NMD activity is attenuated as myoblasts differentiate to myotubes. During myogenesis, gene expression can be downregulated by NMD or by a related pathway, Staufen-mediated mRNA decay (SMD). SMD is increased in myoblasts due to the upregulation of the STAU1 (Staufen homolog) protein, which binds its cognate sites in the 3′ UTR of target mRNAs [111]. STAU1 competes with UPF1 for binding to UPF2, which functions in both NMD and SMD [112, 113]. This competition leads to inhibition of UPF2-dependent NMD and increased expression of the NMD target myogenin that promotes myogenesis [112].

Interactions of cells with external factors such as viruses can also be modulated by NMD. Robust NMD activity targets certain viral RNAs harboring NMD-inducing features to suppress expression of viral proteins and limit viral titer in host cells [114, 115]. However, some viruses possess mechanisms to co-opt the NMD process for their own benefit. For example, it has been found that the RNA-binding proteins tax and rex, expressed by the human T-cell leukemia virus type-1 (HTLV-1), stabilize both viral RNAs and host RNAs that would normally be targets for NMD [116, 117]. An element in the 3′ UTR of the *Rous sarcoma virus* also renders the viral RNA insensitive to host NMD, possibly by inhibiting the capacity of UPF1 to initiate NMD [118, 119]. As another example, hepatitis C infection triggers inactivation of NMD by binding and sequestering WIBG/PYM, a protein required for recycling of the EJC [120].

Recent studies suggest that NMD controls not only the levels of mRNAs, but also that of long non-coding RNAs (lncRNAs). While the majority of the genome is transcribed into RNA, only about 2% of the genome has been shown to code for proteins [121, 122]. LncRNAs are a prominent class of RNA molecules that have important roles in cellular processes, including modifying chromatin, regulating transcription, altering mRNA stability, and influencing translation [123, 124]. A subset of lncRNAs have been shown to be associated with the translation machinery—sometimes producing detectable micropeptides—and about 17% of lncRNAs were found to be targets of NMD [125–130]. While the biological significance of this regulation remains to be defined, the fact that so many lncRNA transcripts are targeted by NMD suggests that NMD plays a central role in regulating the functions of lncRNAs and their corresponding micropeptide products.

## Dynamic regulation of NMD during cellular responses to stress

Cellular stress activates widespread changes in gene expression that allow cells to adapt to challenging conditions. One mechanism that enables this response is the inhibition of NMD (Table 1). Cellular stresses such as amino acid deprivation, hypoxia and endoplasmic reticulum (ER) stress induce phosphorylation of the translation initiation factor eIF2α, which in turn causes NMD repression and the stabilization and increased expression of critical stress response factors such as ATF4, ATF3, CHOP, and IRE1α [70, 73, 131–136]. NMD is also attenuated in response to an increase in intracellular calcium levels as well as persistent DNA damage [137 AN and ZY, unpublished]. By controlling the expression of specific genes, this dynamic regulation of NMD serves as an adaptive response to cope with cellular stress and promote survival. When the environmental insults are too severe, NMD also contributes to apoptosis. An early event during apoptosis is the cleavage of UPF1, which generates a dominant negative peptide fragment that stifles NMD activity [138]. The resulting reduction in NMD activity allows for the upregulation of several pro-apoptotic NMD target genes including GADD45α, GADD45β, BAK1, GAS5, DAP3, and DUSTP2, leading to cell death [138, 139]. GADD45α, which acts in the MAP kinase pathway, has also been proposed to be the key target that triggers apoptosis when NMD is disrupted in the absence of exogenous stress [140].

The observations that NMD is suppressed in response to a number of cellular stresses raises the question of how abnormal RNAs—which are often generated during gene expression—are dealt with during intervals of reduced NMD activity. One possibility is that the benefits of the expression of stress response genes after NMD attenuation outweigh the risks of the lack of RNA surveillance. It is also possible that cells retain residual NMD activity after stress, which is sufficient for RNA surveillance. During these intervals of low NMD activity, the activation of an autoregulatory circuit that leads to increased mRNA stability of NMD factors—which are normally targeted by NMD—rapidly restores NMD activity to appropriate levels once cellular conditions improve [74, 91, 141]. The discovery of alternative branches of the NMD pathway that are apparently independent of UPF2, UPF3, or the EJC introduces the possibility that when one branch of NMD is suppressed other branches still remain active and degrade aberrant transcripts [46, 142, 143]. In support of this idea, the activity of the UPF2-dependent branch of NMD is diminished during myogenesis but an alternative, UPF2-independent branch is stimulated, allowing both for increased expression of the NMD target myogenin and continued degradation of mutant mRNA transcripts [112]. An additional mechanism to cope with reduced NMD activity is autophagy, which purges cells of the mutated, misfolded, and aggregated proteins that accumulate in NMD-deficient cells [144].

## NMD and human disease

Nonsense-mediated RNA decay and its regulation influence the development of human disease. While some disease phenotypes are exacerbated by the effects of NMD, others are suppressed by them, making NMD a “double-edged sword”. One example where NMD contributes to disease is β-thalassemia, which is often caused by mutations in the β-globin gene that generate a nonsense mRNA. Most recessive forms of β-thalassemia result from nonsense mutations in the first or second exon of the β-globin gene, with the corresponding mRNAs being targeted for degradation by NMD [145–147]. In these cases, the unaffected allele is still able to be expressed but the amount of protein produced is unable to compensate for loss of function of the mutant allele. Mutations that occur in the final exon of β-globin evade degradation and consequently are translated normally. However, the resulting truncated proteins have dominant negative activity that interferes with normal hemoglobin function [145].

Numerous other genetic diseases, including cystic fibrosis, polycystic kidney disease, and muscular dystrophy, are also caused by PTCs that trigger NMD of target mRNAs [145, 147]. Interestingly, different subtypes of muscular dystrophy can result from mis-expression of distinct genes that are associated with NMD. Duchenne’s muscular dystrophy results from loss of function of dystrophin, which can occur when mutations in the gene generate a PTC that targets the transcript for NMD. Facioscapulohumeral muscular dystrophy results from the misexpression of the DUX4 transcription factor in muscle. DUX4 is normally a substrate for NMD, but its misexpression in muscle leads to the inhibition of NMD, resulting in a regulatory feedback loop that further stabilizes the DUX4 transcript, leading to cellular toxicity [148].

Certain neurodevelopmental disorders are closely connected with dysregulation of NMD. Mutations in the NMD factor UPF3B have been found to cause syndromic and nonsyndromic intellectual disability (ID) [101, 102, 104, 106]. UPF3B mutations are also associated with a spectrum of disorders including attention-deficit hyperactivity disorder, autism and schizophrenia [102–104]. Dysregulation of other NMD factors such as UPF2 and SMG6, is also associated with various forms of ID [102].

Aberrant NMD also is associated with inflammation and cancer. Deletion of one allele of the NMD kinase SMG1 in a mouse model results in chronic inflammation as well as cancer predisposition [3]. Mutations in UPF1 have been identified in inflammatory myofibroblastic tumors (IMT) [149]. In IMT, decreased NMD function leads to increased expression of the transcript for the NIK protein kinase, which activates the NFkB pathway and promotes cytokine expression and inflammatory infiltrates [149]. Inhibition of NMD can cause chronic activation of the immune response, leading to autoimmunity [150]. Loss of function or overexpression of NMD factors have also been found to be associated with several other cancer types, including pancreatic cancer and neuroblastoma [151–154]. Deregulation of NMD contributes to tumorigenesis likely due to aberrant expression of oncogenes and tumor suppressor genes with PTCs [151, 155, 156].

Although decreased NMD efficiency can cause human disease and contribute to the severity of disease phenotypes, NMD inhibition can also be a strategy for disease treatment. Inhibiting NMD may alleviate the symptoms of certain genetic diseases caused by PTCs in a single gene—e.g. β-thalassemia, cystic fibrosis, Hurler’s syndrome, and Duchenne muscular dystrophy—by allowing expression of a mutant protein product that is partially functional [145]. However, this therapeutic strategy is limited by the ability of the truncated proteins to provide sufficient activity to compensate for the loss of function. A more promising solution may be to restore expression of full-length, functional proteins by combined treatment of NMD inhibitors (to stabilize nonsense transcripts) with drugs that allow stop-codon read-through. This strategy has been successfully used to restore full-length, functional proteins in a model of Hurler’s syndrome and in cancer cells with nonsense mutations in the p53 gene [157, 158]. A recent modification of this potential therapeutic strategy uses antisense oligonucleotides (ASOs), which are showing increasing promise in clinical trials, rather than small-molecule drugs to repress NMD activity, thereby expanding the repertoire of potential NMD-targeted therapeutic strategies [159]. Interestingly, increasing NMD activity, such as by overexpressing UPF1, can alleviate the phenotypes of amyotrophic lateral sclerosis (ALS) in both in vitro and in vivo models. A large fraction of ALS is caused by aberrant expression of TDP43, which deregulates splicing and generates many NMD targets [160, 161]. The observed effects of UPF1 overexpression suggest that NMD enhancers may be effective in treating certain forms of ALS and raise the possibility that a similar principle may apply to other disorders caused by aberrant RNA processing.

Due to the presence of a higher level of nonsense mRNAs caused by mutations and genomic instability in cancer cells, inhibition of NMD may cause accumulation of mutant proteins and activation of the unfolded protein response, leading to heightened cell death. Inhibition of NMD can also promote the expression of novel antigens on tumor cells, due to the translation of nonsense mRNAs generated by frameshift mutations or aberrant splicing [162, 163]. For these reasons, there has been a strong interest in developing small molecules to inhibit NMD activity (Table 2). Compounds such as cycloheximide and puromycin abrogate NMD by inhibiting translation, and other reagents that modify the specificity or efficacy of translation termination—suppressor tRNAs, aminoglycosides, PTC124, amlexanox—are also capable of stabilizing nonsense transcripts [95, 164–170]. Wortmannin and caffeine also inhibit NMD by decreasing SMG1 enzymatic activity, but these inhibitors are limited as tools because they also affect other PI3K family members such as ATM, ATR and DNA-PK [171, 172]. Inhibitors of SMG1 kinase activity with improved potency and selectivity, such as pyrimidine deriviatives, have been identified and shown to substantially diminish UPF1 phosphorylation in vitro and in cells [173]. Recently, other potent small molecule inhibitors selective for SMG1 kinase have been identified to inhibit UPF1 phosphorylation in cells and in mouse tumor xenograft models, where they promote anti-tumor efficacy (JMB, unpublished). Inhibitors to NMD factors other than SMG1 have also been reported. For example, patemine A was found to repress NMD activity by inhibiting the NMD function of eIF4AIII, whereas NMDI-1 blocks NMD by preventing the interaction between SMG5 and UPF1 [23, 174]. NMDI-14 was identified in a computational screen for molecules that physically prevent the interaction of SMG7 with UPF1 [158]. Promisingly, NMDI-1 and particularly NMDI-14 potently repress NMD at low concentrations with minimal cellular toxicity [158, 174]. In addition, the approved drugs 5-azacytidine and cardiac glycosides such as ouabain and digitoxin were recently found to inhibit NMD by upregulating Myc or by increasing intracellular calcium, respectively [137, 175]. These findings point to the potential of NMD-based therapeutic intervention by directly inhibiting NMD factors, or indirectly affecting the cellular microenvironment.

**Table 2:** Small molecules that inhibit NMD efficiency

Compound	Mechanism	References
NMD inhibitors		
PI3K-like kinase inhibitors (e.g. caffeine, wortmannin)	Inhibits SMG1 kinase activity	[171, 172]
NMDI1	Disrupts the interaction between SMG5 and Upf1	[174]
NMDI14	Disrupts the interaction between SMG7 and Upf1	[158]
Patemine A	Inhibits the NMD function of eIF4A3	[23]
5-azacytidine	Promotes expression of c-Myc, which represses NMD	[175]
Cardiac glycosides (e.g. digoxin, ouabain)	Increase cytoplasmic calcium, which represses NMD	[137]
Translation inhibitors		
Cyclohexamide	Inhibits translation	[95]
Emetine	Inhibits translation	[95]
Puromycin	Inhibits translation	[95]
Anisomycin	Inhibits translation	[95]
Translation modifiers		
Suppressor tRNAs	Change stop codons into amino acid-encoding codons	[164–166]
PTC-124	Promotes stop codon read-through	[167]
Aminoglycosides	Promotes stop codon read-through	[168, 169]
Amlexanox	Promotes stop codon read-through	[170]

## Perspectives

Nonsense-mediated RNA decay, initially discovered as a quality control mechanism that targeted mutant transcripts for degradation, is now widely appreciated as a key mechanism that regulates gene expression. NMD plays a crucial role in multiple cellular processes, including development, differentiation and disease physiology. While the main factors that drive NMD have been identified, many opportunities remain to fill in gaps in our understanding of NMD target selection and its impact on cell biology. A major area of ongoing NMD research will concern the complex regulatory networks that govern NMD activity in developmental and tissue-specific contexts. In addition to uncovering new pathways or processes where NMD is dynamically regulated, putative NMD transcripts must be validated and their effects on cell biology elucidated. Work discussed in this review has begun to address this challenge. The contribution of NMD to disease states, particularly neurological disorders and cancer will constitute another major direction of NMD research. The discovery of novel inhibitors—and potentially also enhancers—of the NMD pathway provide the possibility for therapeutic intervention with genetic diseases, neurological disorders, and cancer. NMD inhibition by chemical or genetic means has been demonstrated to restore expression of proteins in vitro, but the viability of these strategies in vivo—including in humans—remains to be tested. Furthering this promising work is paramount to applying our ever-expanding understanding of NMD to the treatment of human disease.
